# Growth performance and carcass characteristics of bulls fed tannins associated or not with monensin

**DOI:** 10.1093/tas/txae136

**Published:** 2024-09-20

**Authors:** Kaique S Nascimento, Lorena Emily L M Bomfim, Victor R M Couto, Mauricia B Silva, Ana Laura A Lopes, Marcia H M R Fernandes, Marcelo Q Manella, Marcos V C Ferraz Junior, Juliano J R Fernandes

**Affiliations:** Universidade Federal de Goiás, Escola de veterinária e Zootecnia, Goiânia, Goiás, Brazil; Universidade Federal de Goiás, Escola de veterinária e Zootecnia, Goiânia, Goiás, Brazil; Universidade Federal de Goiás, Escola de veterinária e Zootecnia, Goiânia, Goiás, Brazil; Universidade Federal de Goiás, Escola de veterinária e Zootecnia, Goiânia, Goiás, Brazil; Universidade Federal de Goiás, Escola de veterinária e Zootecnia, Goiânia, Goiás, Brazil; Department of Animal Science, UNESP – Universidade Estadual Paulista, Jaboticabal Campus, Brazil; Animal Nutrition, Silvateam, Estância Velha, RS, Brazil; Instituto de Ciências Sociais, Educação e Zootecnia, Universidade Federal do Amazonas, Parintins 69152240, Brazil; Universidade Federal de Goiás, Escola de veterinária e Zootecnia, Goiânia, Goiás, Brazil

**Keywords:** crude protein, feedlot, high-grain diets, monensin, tannin

## Abstract

This study aimed to evaluate the effect of tannin (a mix of hydrolyzable and condensed) and its association with monensin on feed efficiency, growth performance, and carcass of bulls fed high-grain diets with 14% and 13% crude protein (**CP**). Bulls (64 Nellore and 96 crossbred Angus × Nellore; initial body weight (**BW**) = 342 ± 25 kg; 20 ± 1 months) were allocated into 20 pens (8 from the same breed per pen). The treatments were T14 – 1.5 g of tannin/kg of dry matter (**DM**) (Bypro; Silva Team Brasil, Estância Velha, Brazil) in a diet with 14% of CP; M14 – 25 ppm monensin (Rumensin; Elanco Saúde Animal, São Paulo, Brazil) in a diet with 14% of CP; TM14 – 1.5 g of tannin/kg of DM and 25 ppm monensin in a diet with 14% of CP; TM13 – 1.5 g of tannin/kg of DM and 25 ppm monensin in a diet with 13% of CP. Data were analyzed using a randomized block design with pens as experimental units. Supplementation with tannin resulted in greater final BW, average daily gain (**ADG**), and dry matter intake (**DMI**) compared with Monensin (*P* < 0.05). The association between Tannin and Monensin decreased (*P* < 0.05) DMI without affecting growth performance, consequently improving the gain-to-feed ratio compared to the Tannin-alone treatment. When Tannin was combined with Monensin, there was an increase (*P* < 0.05) in net energy for gain and net energy for maintenance compared with Tannin supplementation alone. Bulls supplemented with Tannin in their diet exhibited greater (*P* < 0.05) hot carcass weight, carcass gain, and gluteus medius area compared with those supplemented with Monensin alone. Final BW, ADG, and DMI were lower (*P* < 0.05) when the CP content of the diet was decreased from 14% to 13%. The optimal combination for achieving maximum growth and feed efficiency was observed when bulls were fed with tannin and monensin combined in a diet containing 14% CP.

## Teaser

Explore the synergistic effects of tannin and monensin supplementation on bull growth and carcass traits, uncovering key strategies for improving feed efficiency in beef production.

## Introduction

Feed additives enhance the growth performance, feed efficiency, and health of beef cattle (Ferraz Junior and Carvalho, 2022). Among these additives, monensin, classified as an ionophore, effectively inhibits Gram-positive bacteria (Russell and Strobel, 1989). This inhibition leads to a modification of rumen fermentation by altering the ratio of short-chain fatty acids in the rumen, increasing propionate (Duffield et al., 2012). A prior meta-analysis indicated that monensin has a notable impact on improving feed efficiency and reducing feed intake, although its effect on average daily gain (**ADG**) is relatively mild (Duffield et al., 2012). Furthermore, monensin has been identified as a substance that hinders rumen deamination reactions, resulting in a reduction in rumen protein fermentation (Russell and Strobel, 1989). However, it concurrently enhances total tract protein digestibility (Rogers et al., 1997).

Among natural and alternative additives, tannins are polyphenolic compounds that stand out for inhibiting the development of some undesirable ruminal bacteria (Scalbert, 1991), changing the profile of some ruminal fatty acids (Beauchemin et al., 2007), and decreasing methane production (Naumann et al., 2017). However, the effect of tannin on leveraging feed efficiency and performance of feedlot cattle remains controversial, mainly due to differences in tannin sources and doses (Mezzomo et al., 2015). For instance, previous studies reported an improvement in ADG of animals fed a blend of hydrolyzable and condensed tannin included at 0.3% (Barajas et al., 2012) or 0.6% (Rivera-Méndez et al., 2017) in high-grain diets. Conversely, other studies reported no effect of tannins (hydrolyzable and/or condensed) on ADG of animals fed high-grain (Krueger et al., 2010) or high-forage diet (Aboagye et al., 2018). In this line, Carvalho et al. (2024) observed that 1.5 g condensed tannin/kg of DM in feedlot diets did not significantly affect the overall growth performance of dairy and beef on dairy males on feedlot.

Monensin and tannin affect rumen fermentation and metabolism through diverse mechanisms. Our hypothesis posits that their synergistic association may lead to enhanced ADG and improved feed efficiency, surpassing the effects observed when these additives are employed individually. This study aimed to assess the impact of tannin, either in conjunction with or independently of monensin, on the feed efficiency and growth performance of feedlot cattle fed with high-grain diets. Additionally, this study aimed to investigate the combined utilization of tannin and monensin in diets featuring varying levels of crude protein (**CP**), specifically 14% and 13%.

## Material and Methods

Experimental procedures were approved by the Animal Use Ethics Committee of the Federal University of Goiás, Brazil, protocol no. 056/18.

### Animals, Diets, Sampling, and Data Collection

Sixty-four Nellore and 96 crossbred Angus × Nellore (initial body weight (**BW**) = 342 ± 25 kg; 20 ± 1 months) were allocated into 20 collective pens (5 per treatment) of 77 m² (8 bulls from the same breed per pen), provided with concrete trough (7.7 m of line) and drinker (500-L every 2 pens). Animals were blocked by initial BW and breed. The experimental period consisted of 14 d of adaptation to experimental installations and diet; and 90 d of evaluation of performance. Animals were weighed at the beginning and end of the experiment after a 14-h solid fast.

Treatments consisted of diets with additives monensin (Rumensin; Elanco Saúde Animal) and tannin (a blend of 1/3 of hydrolyzable tannin from chestnut (*Castanea sativa*) and 2/3 of condensed tannin from quebracho (*Schinopsislorentzii*); Bypro (SilvaTeam Brasil), as follow: T14 – 1.5 g of tannin/kg of dry matter (**DM**) (Bypro; Silva Team Brasil) in a diet with 14% of CP; M14 – 25 ppm monensin (Rumensin; Elanco Saúde Animal) in a diet with 14% of CP; TM14 – 1.5 g of tannin/kg of DM and 25 ppm monensin in a diet with 14% of CP; TM13 – 1.5 g of tannin/kg of DM and 25 ppm monensin in a diet with 13% of CP.

Bulls were fed once daily at 0800 hours. Feed was offered ad libitum, with the amount adjusted to ensure that 5% of the feed was left over after each day, and comprised of sugarcane bagasse (98 g/kg DM) as roughage source, and concentrate (902 g/kg DM; Table 1). Orts were collected, weighted, sampled daily, and stored at −20 °C for further chemical analyses.

**Table 1.** Ingredient and chemical composition of diets

	Treatments^a^			
Ingredients (g/kg DM)	M14	TM14	T14	TM13
Sugarcane Bagasse	98	98	98	98
Cracked corn	753.3	752	752.1	800.1
Soybean meal	122.5	122.5	122.5	70.7
Urea	7.1	7.1	7.1	10.8
Supplement^b^	18.8	18.8	18.8	18.8
Monensin^c^	0.1	0.1	-	0.1
Tannin^d^	—	1.5	1.5	1.5
*Chemical composition* ^e^ *(g/kg DM)*				
DM (g/kg as fed)	651.4	641.9	633.4	641.4
Crude protein	146.7	142.9	148.7	133.3
Ether extract	29.3	30.1	29.9	32.5
aNDF	177.1	166.2	155.9	172.7
ADF	80.4	69.5	68.4	73.5
Organic matter	957.5	961.2	967.1	960.3
Ash	42.5	38.8	32.9	39.7
NEm, Mcal/kg^f^	1.96	1.98	2.00	1.97
NEg, Mcal/kg^f^	1.32	1.33	1.35	1.32

^a^M14: 140 g crude protein (CP)/kg dry matter (DM), and 25 ppm monensin; TM14: 140 g CP/kg DM, 25 ppm monensin, and 1.5 g/kg tannin; T14: 140 g CP/kg DM and 1.5 g/kg tannin; TM13: 130 g CP/kg DM, 25 ppm monensin, and 1.5 g/kg tannin.

^b^Supplement contained in 1,000 g: calcium: 185 g; phosphorus: 60 g; sodium: 111 g; magnesium: 10 g; sulfur: 18 g; copper: 1,000 mg; manganese: 800 mg; zinc: 3,600 mg; cobalt: 75 mg; iodine: 75 mg; selenium: 11 mg; flour: 600 mg.

^c^25 mg/kg DM, Monensina (Rumensin); ElancoSaúde Animal.

^d^Taninns (Bypro); SilvaTeam Brasil.

^e^DM = dry matter; aNDFom = neutral detergent fiber with heat stable amylase and exclusive of ash, ADFom = acid detergent fiber exclusive of ash.

^f^NEm = net energy for maintenance. NEg = net energy for gain. Estimated from feed composition (NRC, 1996).

At the end of the experimental period, in vivo images obtained with ultrasound (Aloka 500V, Hitachi Aloka Medical, Ltd, Toquio, Japan) between the left side of the 12th and 13th ribs were performed to evaluate12th-rib fat and LM area. Rump fat thickness and *Gluteus medius* area were determined at the intersection of the *Gluteus medius* and *Biceps femoris* muscles, located between the ilium and ischium bones (Realini et al., 2001). Images were collected by a certified Ultrasound Guideline Technician Council and images were interpreted using Biosoft Toolbox II software (Biotronics, Inc., Ames, IA, USA).

Animals were slaughtered in a commercial slaughterhouse. Immediately after slaughter, hot carcass weight (kg) was determined to calculate the carcass dressing weight relative to the final BW.

### Chemical Analyses and Estimation of Dietary Net Energy

Samples of feed ingredients and orts were dried at 55 °C for 72 h and ground in a Wiley mill (Thomas Scientific, Swedesboro, NJ, USA) to pass through a 1-mm screen and analyzed according to AOAC Methods (2000) as follows DM (method 930.15); ash (method 942.05); CP (method 976.05); ether extract (method 920.39), acid detergent fiber exclusive of ash (**ADFom**: method 973.18). Determination of a neutral detergent fiber (**aNDFom**) was performed according to Mertens (2002), using thermostable amylase (Liquozyme), without sodium sulfite, and exclusive of ash.

Daily energy gain (**EG**; Mcal/d) was estimated by the equation: EG = (0.0557 × BW^0.075^) × ADG^1.097^, where BW is the mean BW (kg; NRC, 1984). Maintenance energy (**ME**; Mcal/d) was estimated as follows: ME = 0.077 × BW^0.75^ (Lofgreen and Garret, 1968). Dietary energy for maintenance (**NEm**) and gain (**NEg**) was calculated as follows (Zinn and Shen, 1998): NEm = (−*b* − ((*b*²) − (4*ac*))^0.5^))/2, where *a* = −0.41 × ME, *b* = 0.877 × ME + 0.41 × DMI + EG, and *c* = −0.877 × DMI; NEg = 0.877 × DMI, in which **DMI** was dry matter intake.

### Experimental Design and Statistical Analysis

Data were analyzed using a randomized block design with pens as experimental units (5 replicates per treatment). Animals were blocked by weight and breed (Nellore or crossbred Angus × Nellore). All statistical procedures were performed using SAS software (SAS Institute Inc., Cary, NC). Mean comparisons were performed using contrasts: 1) tannin vs. monensin, 2) monensin vs. the associative effect of monensin and tannin, 3) tannin vs. the associative effect of monensin and tannin, and 4) the associative effect of monensin and tannin with 13% or 14% CP. Significance was declared at *P* ≤ 0.05.

## Results

Supplementation with tannin resulted in greater final BW, ADG, and DMI compared with monensin (*P* < 0.05; Table 2). The association between tannin and monensin decreased (*P* < 0.05) DMI without affecting growth performance, consequently improving the gain-to-feed ratio (**G:F**) compared to the tannin-alone treatment. No differences (*P* > 0.05) were observed between tannin and monensin supplementation regarding energy variables. However, when tannin was combined with monensin, there was an increase (*P* < 0.05) in NEg and NEm compared to Tannin supplementation alone. This combination resulted in a notable 5% increase in the observed-to-expected ratio of NEm and NEg in the assessment.

**Table 2.** Performance and carcass traits of bulls fed monensin and tannins

Item	Treatments^a^				SEM	*P*-value			
	M14	T14	TM14	TM13		M14 vs. T14	M14 vs. TM14	T14 vs. TM14	TM14 vs. TM13
*Feedlot performance*									
Initial BW^b^, kg	343.15	343.4	342.8	341.98	13.85	0.88	0.83	0.72	0.62
Final BW^b^, kg	526.16	545.33	540.71	527.98	29.98	<0.01	<0.01	0.33	0.02
ADG^b^, kg/d	1.73	1.91	1.88	1.76	0.24	<0.01	<0.01	0.45	0.03
DMI^b^, kg/d	9.76	10.92	10.28	9.78	0.71	<0.01	0.02	<0.01	0.02
DMI^b^, % BW	2.25	2.46	2.34	2.26	0.07	<0.01	0.05	<0.01	0.06
G:F^b^, g/kg	177.8	175	182.5	180.2	11.08	0.39	0.16	0.03	0.47
*Energy*									
NEm^b^, Mcal/kg	2.05	2.00	2.08	2.06	0.09	0.12	0.28	0.02	0.60
NEg^b^, Mcal/kg	1.39	1.35	1.41	1.40	0.08	0.08	0.34	0.01	0.66
Observed:expected NEm	1.03	1.01	1.05	1.04	0.05	0.12	0.29	0.02	0.50
Observed:expected NEg	1.05	1.01	1.06	1.05	0.06	0.09	0.40	0.02	0.67
*Carcass traits*									
HCW^b^, kg	291.87	300.74	300.68	292.76	14.62	0.05	0.05	0.98	0.07
Carcass gain, kg/d	1.14	1.22	1.22	1.16	0.12	0.04	0.02	0.95	0.09
Dressing percentage, %	55.42	55.21	55.6	55.42	0.52	0.69	0.74	0.47	0.74
LM area^b^, cm²	87.2	88.77	92.34	89.58	4.36	0.57	0.08	0.21	0.33
12th-rib fat, mm	5.09	5.18	5.43	5.22	0.91	0.82	0.40	0.54	0.60
Rump fat thickness, mm	5.76	5.62	5.81	5.55	0.86	0.67	0.87	0.56	0.42
Gluteus medius area, cm²	92.79	99.47	98.88	97.75	6.91	0.04	0.06	0.84	0.71

^a^M14: 140 g crude protein (CP)/kg dry matter (DM) and 25 ppm monensin; TM14: 140 g CP/kg DM, 25 ppm monensin, and 1.5 g/kg tannin; T14: 140 g CP/kg DM and 1.5 g/kg tannin; TM13: 130 g CP/kg DM, 25 ppm monensin, and 1.5 g/kg tannin.

^b^BW, body weigh; ADG, average daily gain; DMI, dry matter intake; G:F, gain to feed ration; NEm, net energy for maintenance; NEg, net energy for gain; HCW, hot carcass weight; LM, longissimus muscle.

Bulls supplemented with tannin in their diet exhibited significantly greater (*P* < 0.05) hot carcass weight, carcass gain, and gluteus medius area compared to those supplemented with monensin alone. The combined supplementation of tannin with monensin increased (*P* > 0.05) hot carcass weight (*P* = 0.05) and carcass gain (0.02, respectively) compared to monensin alone. The other carcass parameters were not affected by treatments.

Final BW, ADG, and DMI were lower (*P* < 0.05) when the CP content of the diet was decreased from 14% to 13%. Energy and carcass variables were not affected (*P* > 0.05) by the CP level in the diet.

## Discussion

The primary hypothesis of this study posited that the combination of tannin and monensin, employed as feed additives, could enhance the growth performance of bulls fed a high-gain diet. This hypothesis was partially confirmed. The co-administration of both feed additives resulted in increased final BW and ADG compared with the use of monensin alone. However, the G:F was found to be similar to the monensin-only group. On the other hand, when tannin was paired with monensin, final BW and ADG were comparable to tannin alone, but the G:F ratio exhibited an improvement in the tannin and monensin combination. This outcome underscores the pivotal role of DMI. When considering all the results collectively, it became evident that monensin played a role in decreasing DMI in this study. The impact of monensin on reducing DMI is well-documented (Duffield et al., 2012), yet the precise reasons for this occurrence remain relatively obscure.

In contrast, tannin increased DMI compared with monensin. This result was corroborated by previous studies that used a similar blend of tannins from Bypro (Barajas et al., 2011; Tabke et al., 2017; Rivera-Méndez et al., 2017). The phenolic group of tannins is an excellent hydrogen donor, enabling tannins to bind other molecules, such as protein, and, consequently, reducing the digestibility of DM, organic matter, and protein (Koenig and Beauchemin, 2018; Norris et al., 2020). Hence, the reduced digestibility might induce lower NEm and NEg of diets with tannins (Galyean and Defoor, 2003), as observed in the current study, which may increase intake to supply energy requirements for gain. In this study, the greater DMI was associated with greater ADG and final BW. Nonetheless, the combination of tannins and monensin improved feed efficiency compared with monensin and tannin alone, indicating a synergistic effect between tannin and monensin, in which monensin reduced DM and Tannin increased ADG.

Koenig and Beauchemin (2018) observed that the use of tannins decreased the ruminal degradability of the protein. The supply of degradable protein in the rumen (RDP) must be balanced with the ruminal availability of energy to maximize microbial protein production. Soybean meal is the main source of protein in Brazilian feedlots with high ruminal digestibility (Pinto and Millen, 2019). When fed to bulls, soybean meal together with urea may increase rumen ammonia concentrations, increasing its absorption via the rumen wall and consequently excretion via urine, resulting in low efficiency of N use (Calsamiglia et al., 2010). We speculated that the use of tannins in association with monensin (TM14) may have provided an increase in the efficiency of nitrogen use, making the feed efficiency higher than T14.

Tannins modify the protein metabolism of ruminants by increasing microbial protein production through more efficient nutrient partitioning. Bulls fed diets with low tannin levels show higher performance due to a greater flow of essential amino acids to the small intestine. This results from more efficient microbial protein production and an increased amount of true protein (Makkar et al., 1998). This is supported by the finding that bulls fed TM13 had lower growth performance compared to those fed TM14. Therefore, protein levels should not be reduced to 13% in diets containing tannins and monensin if the goal is maximum growth.

### Conclusion

Monensin significantly decreased DMI, leading to improved feed efficiency. Conversely, tannin exhibited the opposite effect, elevating both DMI and maximizing growth. The optimal combination for achieving maximum growth and feed efficiency was observed when bulls were fed a blend of condensed and hydrolyzable tannins in conjunction with monensin in a diet containing 14% CP. However, when the CP content of the diet was reduced to 13%, a decrease in growth performance was noted.
